# Effects of aerobic and resistance exercises on circulating apelin-12 and apelin-36 concentrations in obese middle-aged women: a randomized controlled trial

**DOI:** 10.1186/s12905-019-0722-5

**Published:** 2019-01-29

**Authors:** Sun-Hwa Jang, Il-Young Paik, Jae-Hoon Ryu, Tae-Hyung Lee, Dae-Eun Kim

**Affiliations:** Exercise Physiology Laboratory, Department of Physical Education, Yonsei University, Seoul, 03722 South Korea

**Keywords:** Obese middle-aged women, Apelin-12, Apelin-36, Aerobic exercise, Resistance exercise

## Abstract

**Background:**

The risk for obesity-related diseases increases with the prevalence of obesity. In obesity, adipokines secreted from adipose tissue induce inflammation, causing adverse effects. Recently, adipokines such as apelin, visfatin, and chemerin have been studied. Long-term resistance training improves health in middle-aged women by improving metabolic risk factors, body composition, and muscle strength. However, there is still a lack of evidence on the association of apelin concentration with different exercise types in middle-aged obese women This study aimed to investigate the effects of 8 weeks of aerobic and resistance exercises on apelin-12 and apelin-36 levels and thereby verify the effects of different exercise types in obese, middle-aged women.

**Methods:**

Participants were middle-aged women aged 50–61 years, with no experience of systematic exercise in the last 6 months, and met the WHO obesity criteria for the Asia-Pacific region of waist circumference ≥ 80 cm and body fat percentage ≥ 30%. Subjects were selected and allocated to the aerobic exercise, resistance exercise, or no exercise group by block randomization. Body weight, body fat, and body mass index were measured by bioelectrical impedance analysis. Analysis of variance, the t-test, and Tukey’s post-hoc test were performed.

**Results:**

A total of 24 participants were selected with eight participants in each group. Both aerobic and resistance exercises were effective in altering the physical composition, showing significant decreases in weight, waist circumference, BMI, and body fat. The aerobic and resistance exercise group showed a significant, positive change in apelin-12 levels.

**Conclusions:**

In obese individuals, aerobic and resistance exercise were effective in improving obesity and reducing blood apelin-12 concentration, which is closely correlated with indicators of metabolic syndrome.

Future research should focus on comparing the response of apelin to exercise in obese subjects treated with only dietary control and the response in the obese subjects of different ages and sex.

**Trial registration:**

No. 1040917–201,506-BR-153-04, Clinical Research Information Service (CRIS), Republic of Korea (05 October 2018, retrospectively registered).

## Background

Recent societal advances, medical progress, and abundance of food have led to improvements in living environment, longer life expectancy, and increases in the proportion of the middle-aged population. However, there has been a decreasing trend in healthy life expectancy due to disease or injury [1]. For women in particular, if health is not properly managed when entering the middle age, autonomic imbalance, reduced energy expenditure, reduced muscle mass, and increased abdominal obesity may occur [2]. Obesity, which is a prevalent cause of disease, refers to the state of excessive fat mass as a result of an imbalance between energy intake and expenditure due to lack of physical activity, overeating, or genetic factors [3]. Obesity increases with age, with a prevalence of 22.4% for 20–29-year-olds, 33.7% for 40–49-year-olds, and 36.3% for 60–69-year-olds, and is a risk factor for various diseases [4]. Obesity metabolically increases the risk of coronary artery disease, cardiovascular disease, and chronic illness and, in particular, is a risk factor for type 2 diabetes and metabolic syndrome by increasing insulin resistance [5, 6]. Moreover, excessive abdominal and visceral adipose tissue causes metabolic syndrome and, when neglected for a long time, increases the risk of various complications [7]. In women, body fat increases rapidly and the basal metabolic rate decreases after menopause, which leads to increased prevalence of metabolic syndrome [8, 9]. Adipokines are proteins secreted by adipose tissue. Changes in adipokine secretion have been observed in obesity, type 2 diabetes, and metabolic syndrome, and these proteins play a role in lipogenesis and lipolysis via glucose absorption and fat [10]. These hormones are also related to appetite regulation, energy homeostasis, increased blood pressure, inflammation, and immune activity [11]. Adipokines secreted in obese individuals have negative effects on the body by promoting insulin resistance and inflammation [12]. Adipokines include leptin and adiponectin [13]. New adipokines, such as apelin, visfatin, and chemerin, have recently been discovered in ongoing research [14].

Apelin is known to be a peptide ligand of the G-protein couple receptor APJ [15], produced and secreted by adipose tissue and cells in humans and rodents [16], and affects various physiological processes, including modulating cardiovascular function, glucose homeostasis, promoting insulin sensitivity, modulating energy metabolism, and lipogenesis and lipolysis [17]. In particular, in lipid metabolism, apelin promotes lipolysis via a pathway involving adenosine monophosphate-activated protein kinase, Gq, and Gi [18]. At the same time, insulin is an important modulator of apelin expression and secretion, and high concentrations of apelin are observed in hyperinsulinemia, indicating a correlation between apelin and insulin [19]. Recent studies have found high concentrations of apelin in obese individuals and in type 2 diabetes patients, while showing correlations of apelin with homeostatic model assessment-insulin resistance, body mass index, total cholesterol, low-density lipoprotein cholesterol, and insulin [20, 21]. Based on this evidence, apelin appears be a relevant indicator for metabolic syndrome and related to type 2 diabetes [22].

The American College of Sports Medicine [23] recommends at least 30 min of moderate physical activity per week, if possible, and proposes that moderate and vigorous physical activities reduce the risk factors for metabolic syndrome [24]. Long-term resistance training has been reported to improve health in middle-aged women, by improving metabolic risk factors, body composition, and muscle strength [25]. Studies have shown a decrease in apelin after 8 weeks of aerobic training at 60–70% maximum heart rate (HRmax) in middle-aged obese individuals [26] and after 6 months of resistance training at 60–80% 1 repetition maximum (1RM) in type 2 diabetes patients [27]. In addition, recent research has shown that apelin is associated with glucose concentration, reduced triglycerides, and rapid lipolysis after exercise or continual physical activity and that this has a positive effect on prevention of chronic illness [28]. Nevertheless, there is still a lack of evidence on the association of apelin concentration with different exercise types in middle-aged obese women. Therefore, in this study, we aimed to observe apelin concentration following aerobic or resistance exercise in middle-aged obese women. The purpose of this study was to investigate the association of apelin concentration with aerobic exercise and resistance exercise in middle-aged obese women. Moreover, we aimed to answer the following questions: Is there any difference in the apelin concentration change after the exercise by each group? Is weight loss through exercise correlated with blood apelin and body composition? To obtain the answer to questions, we analyze and compare baseline and blood analysis indicators.

## Methods

This randomized controlled trial was conducted for a period of 4 months. One month prior to the study, the subjects were selected for the study, and blood test was performed. The identified subjects were divided into 8 weeks of aerobic exercise and resistance exercise. After the test, post-test was carried out in the same manner as the pre-test.

### Subjects

In this study, the subjects were middle-class, middle-aged women (aged 50–61 years) who were homemakers with no other occupation and residents of Seodaemun District in Seoul where the exercise experiment was conducted. The study selected subjects with no experience of systematic exercise in the last 6 months and met the World health Organization obesity criteria for the Asia-Pacific region of waist circumference (WC) ≥80 cm and body fat percentage ≥ 30%. The following people were excluded from the study: those taking medication for chronic disease (such as diabetes, rheumatism, or arthritis); those who have been diagnosed or currently have angina pectoris, myocardial infarction, or brain disease; and those who cannot participate in the experiment due to physical, mental, or physiological impairment. The subjects were randomly selected except for those who received voluntary applications and did not meet the required fulfillment requirements. Finally, 24 participants were enrolled. To balance the number of subjects per group, a random number table was created and the subjects were assigned by block randomization. The subjects were divided into the aerobic exercise group (AEG), the resistance exercise group (REG), and the control group (CG), with 8 subjects in each group. The physical characteristics of the subjects are shown in Table 1.

**Table 1:** Physical characteristics

	AEG (*n* = 8)	REG (*n* = 8)	CG (*n* = 8)
Age (years)	56.62 ± 4.02	54.62 ± 4.02	55.14 ± 3.27
Height (cm)	159.31 ± 3.23	157.82 ± 6.52	154.42 ± 2.58
Body weight (kg)	66.06 ± 5.76	63.95 ± 6.79	59.82 ± 4.25
Waist circumference (cm)	90.62 ± 4.61	91.81 ± 8.74	87.64 ± 5.4
BMI (kg/m^2^)	26.02 ± 2.02	25.73 ± 3.12	25.1 ± 1.69
Body fat (%)	36.72 ± 3.39	37.52 ± 4.34	38.17 ± 3.05

Values are given as means ± S.E

*AEG* aerobic exercise group, *BMI* body mass index, *CG* control group, *REG* resistance exercise group

Subjects abstained from food and drink for at least 12 h prior to blood sample collection. Alcohol intake was strictly forbidden from the day before the pre-test to after the post-test. Besides alcohol, there were no other dietary restrictions, allowing the subjects to maintain their normal diet. Subjects were restricted from exercising, starting from 24 h prior to measurements, while they were also instructed not to eat or drink for at least 12 h prior to the test. The subjects were also instructed to use the restroom to urinate or defecate between 30 min and immediately before the measurement was taken.

Before starting the study, we obtained approval from the Yonsei University Institutional Review Board (approval no. 1040917–201,506-BR-153-03). The study adhered to CONSORT guidelines. Participants were fully explained of the study’s purpose and experiments. Subsequently, they provided written consent, voluntarily participated in blood collection, and disclosed relevant personal information.

## Experimental procedure

### Basic tests

As part of the basic testing for all subjects, height was measured using the semi-automatic anthropometer BSM330 (Bio-space, Korea) and WC was measured using a tape measure (Seca200, Germany), with the measurement made at the approximate midpoint between the lower margin of the last rib and the top of the iliac crest after exhaling while in upright position, as indicated in the WHO guidelines for Asia and Western Pacific region [29]. Body weight, body fat, and BMI (calculated using the formula weight (kg) ÷ {height (m)}2) were measured by bioelectrical impedance analysis using an Inbody720 (Bio-space, Korea), a tetrapolar 8-point tactile electrode system. The subjects were prohibited from wearing any metallic accessories that may interfere with electrical impedance during the bioelectrical impedance analysis and the analysis was performed with all subjects barefooted and wearing the same clothing provided.

Tests were conducted after the subjects performed simple stretching and warm-up exercise.

Moreover, all pre-test and post-test measurements were taken early in the morning before they started any other activities, within 1 h from waking up after at least 8 h of sleep. To meet the measurement conditions, the subjects were provided with light clothing for the measurement.

### Aerobic exercise and resistance exercise

All subjects participated in training 4 times per week for 8 weeks. For the AEG, a Polar heart rate monitor (Polar Electro, Finland) was used during training; each subject’s percent heart rate reserve (%HRR) was calculated using the Karvonen formula, and the intensity was maintained at 60–75% HRR for 50 min [30]. For the REG, subjects performed upper and lower body exercises for 50 min at an intensity of 60–70% 1 RM (repetition maximum) using a weight machine (Technogym, Italy) with a low risk of injury (Table 2).

**Table 2:** Exercise program

	Exercise type	Intensity	Time
Aerobic exercise group	Treadmill running	60–70% HRR	50
Resistance exercise group	Chest press, lat pull down, arm curl, leg press, shoulder press, pectoral, leg curl, leg extension	60–70% 1RM	50

*HRR* Heart rate reserve, *RM* Repetition maximum

### Blood tests

Blood was collected only twice: once before and once after the 8-week exercise program. Under the guidance of a doctor, a specialist nurse collected blood with the patient at rest, taking care to ensure the patients’ safety and following proper hygiene procedures. To measure apelin-12 and apelin-36 concentrations, the blood sample was mixed thoroughly and centrifuged for 10 min at 3000 rpm, and the supernatant was collected and moved to a microtube. Using a microplate reader (VERSA Max, Molecular device, USA) and a human, rat, mouse apelin-12 and apelin-36 extraction-free EIA kit, the sample was analyzed by enzyme-linked immunosorbent assay.

### Data processing

For statistical analysis, SPSS/PC+ Ver. 21.0 K was used. Descriptive statistics (mean and standard deviation) was calculated. A two-way repeated analysis of variance (ANOVA) was used to test differences between groups and before and after training. In the case of a significant interaction, one-way ANOVA and t-test were employed to examine the simple main effects of the independent variables. Then, Tukey’s post-hoc test were performed. We used a statistical significance level of *p* < .05.

## Results

### Changes in physical composition

The effects of 8 weeks of training on physical composition are shown in Table 3. In the two-way ANOVA to examine differences in weight, WC, BMI, and body fat percentage, there was a statistically significant interaction between time and group (*p* < .001). In paired-samples t-tests to examine the effects of time (pre- and post-training), the AEG group had significant decreases in weight (*p* < .001), WC (*p* < .001), BMI (*p* < .001), and body fat percentage (*p* < .01), and the REG also showed significant decreases in weight (*p* < .001), WC (*p* < .001), BMI (*p* < .001), and body fat percentage (*p* < .001). However, the CG did not show any statistically significant changes. There were no statistically significant differences between the groups in terms of weight (*p* < .725), WC (*p* < .845), BMI (*p* < .905), and body fat (*p* < .205).

**Table 3:** Changes in physical composition after 8 weeks of aerobic or resistance exercise

	Group	Pre-training	Post-training		*F*	*P-value*
Body weight (kg)	AEG	66.06 ± 6.16	59.15 ± 4.76^a^	Time	95.452	.000***
	REG	63.95 ± 7.25	58.52 ± 6.09^a^	Group	.327	.725
	CG	60.21 ± 4.39	60.53 ± 4.71	Time×Group	28.991	.000***
Waist circumference (cm)	AEG	90.62 ± 4.93	82.37 ± 6.53^a^	Time	62.453	.000***
	REG	91.81 ± 9.35	82.77 ± 8.80^a^	Group	.170	.845
	CG	88.50 ± 5.80	88.50 ± 6.32	Time×Group	15.71	.000***
BMI (kg/m^2^)	AEG	26.02 ± 2.16	23.31 ± 1.84^a^	Time	96.339	.000***
	REG	25.73 ± 3.33	23.57 ± 2.91^a^	Group	.100	.905
	CG	25.60 ± 2.42	25.18 ± 1.92	Time×Group	29.435	.000***
Body fat percentage (%)	AEG	36.72 ± 3.62	32.70 ± 5.24^a^	Time	63.792	.000***
	REG	37.57 ± 4.54	33.86 ± 4.38^a^	Group	1.709	.205
	CG	38.45 ± 3.15	38.18 ± 3.26	Time×Group	13.036	.000***

Values are given as means ± S.E

*AEG* aerobic exercise group, *BMI* body mass index, *CG* control group, *REG* resistance exercise group. ^a^Significant effect in pre- and post-test among the group (*p* < .05), ***p < .001

#### Changes in apelin-12 and apelin-36 concentrations

The effects of 8 weeks of training on apelin concentration are shown in Table 3. In the two-way ANOVAs to examine changes in apelin-12 and apelin-36 concentrations, there was a statistically significant interaction between time and group for apelin-12 (*p* < .01; *p* < .001), but there was no significant interaction for apelin-36. In a paired-sample t-test to examine the effects of time (pre- and post-training), the AEG showed a significant decrease in apelin-12 (*p* < .05), the REG showed a significant decrease in apelin-12 (*p* < .01), and the CG showed a significant increase in apelin-12 (*p* < .05). There were no statistically significant differences between the groups.

## Discussion

This study investigated the effects of aerobic and resistance training on physical composition and apelin-12 and apelin-36 in middle-aged women of 50–61 years without prior exercise experience. In terms of physical composition, the AEG and REG both showed significant decreases in body weight, WC, BMI, and body fat percentage.

### Effects on physical composition

With gradual advances in modern medical science, participation in regular moderate-to-vigorous physical activities or exercises is recommended to improve personal and public health and physical strength and to reduce the rate of early mortality [31]. In addition, at least 30 min of moderate-to-vigorous aerobic exercise per day for 5 days a week and resistance exercise involving major muscle groups 2–3 days per week help prevent chronic disease and extend health lifespan in adults [32]. Increasing age, ample food intake, reduced physical activity, and lack of exercise can increase the risk for obesity. In addition, the rapid increase in the number of patients with diabetes, metabolic syndrome, musculoskeletal disorders, and cardiovascular disorders are considered major public health problems [33]. Thus, to resolve these problems, the American College of Sports Medicine has presented guidelines advising healthy adults under 65 years old or patients with chronic disease aged 50–64 years to participate in moderate aerobic exercise 5 days a week or 8–10 types of muscle strengthening exercise twice a week [34]. Meanwhile, for metabolic syndrome caused by obesity or insulin resistance, resistance exercise at 60–70% 1RM and aerobic exercise at 40–60% HRmax have been shown to be most effective [35, 36].

In this study, to investigate the effects of different exercise types on weight loss and apelin levels, subjects were divided into an AEG, REG, and CG and participated in 8 weeks of training. In terms of physical composition, the AEG and REG both showed significant decreases in body weight, WC, BMI, and body fat percentage. These results are consistent with previous studies implementing aerobic or resistance exercise, which also reported positive changes in physical composition [37–39]. Thus, our results are supported by the fact that aerobic and resistance exercises lead to reductions in body fat and fat mass, which is an effect of the hormonal response to exercise, even in middle-aged women, who show low hormone levels and poor physical function. Ho et al. also observed similar results after different types of exercise in 40–66-year-old obese women [40]. In addition, with regard to the decrease in body weight following resistance exercise, obesity-related risk factors were not only prevented, but low intramuscular protein and bone density actually improved, accompanied by an increase in lean body mass. This is thought to be due to changes in circulating growth hormone levels shown by adults in response to resistance exercise, which is also accompanied by a decrease in serum cholesterol after training [41]. The most effective method to correct obesity is body weight regulation through exercise, which can increase energy consumption via lipid oxidation with minimal reduction of lean body mass. While high-intensity resistance exercise is much better at increasing lean body mass, low- and moderate-intensity aerobic exercises have a greater effect on fat loss [42]. Thus, exercise time and intensity should be adjusted for the individual, and we believe that an appropriate combination of aerobic and resistance exercise is most effective for obese women with a high risk of disease (Table 4).

**Table 4:** Changes in apelin-12, 36 after 8 weeks of aerobic or resistance exercise

	Group	Pre-training	Post-training		*F*	*P-value*
Apelin-12 (ng/μm^3^)	AEG	.12 ± .02	.07 ± .31^a^	Time	2.737	.115
	REG	.11 ± .02	.08 ± .02^a^	Group	.720	.498
	CG	.09 ± .04	.13 ± .04^a^	Time×Group	14.044	.000***
Apelin-36 (ng/μm^3^)	AEG	.08 ± .02	.09 ± .02	Time	3.184	.089
	REG	.09 ± .03	.09 ± .03	Group	.646	.534
	CG	.08 ± .05	.12 ± .04	Time×Group	1.551	.235

Values are given as means ± S.E

*AEG* aerobic exercise group, *BMI* body mass index, *CG* control group, *REG* resistance exercise group ^a^Significant effect in pre- and post-test among the group (*p* < .05), ***p < .001

### Changes in apelin-12 and apelin-36 concentrations

Apelin is a recently discovered adipokine that has been reported to improve sugar intake and insulin sensitivity, to promote energy consumption, and to reduce blood pressure, while also being important in obesity-related metabolic disease [17, 43]. The secretion and expression of apelin, which is secreted from the heart, blood vessels, and white adipose tissue, are regulated by insulin; thus, apelin is closely related to metabolic syndrome and other disorders caused by diabetes or insulin resistance [44]. Apelin is expressed by adipose tissue, mediated by insulin, promotes glucose uptake via the PI3K/Akt pathway, improves insulin resistant inflammation, and promotes GLUT4 transport from the cytoplasm [45]. Krist et al. reported that apelin is upregulated in obesity, insulin resistance, diabetes, and hyperlipidemia, and the apelin concentration decreases after weight loss via exercise [46]. In our study, when we measured apelin-12 and apelin-36 concentrations after 8 weeks of exercise, only apelin-12 showed a significant interaction effect between group and time. The AEG and REG both showed significant decreases in apelin-12 following training. This is consistent with another study in which apelin was reduced by 8 weeks of aerobic exercise [26]. Moreover, apelin is also involved in suppressing food intake, and one study reported changes in apelin concentration after weight loss via dietary regulation [47]. Apelin-12 also decreased after resistance exercise. Apelin-12, − 13 and − 36 have been reported to show 100% cross-reactivity, but given that most previous studies have investigated changes in apelin-12, it is thought that apelin-12 responds with more sensitivity in humans, and this supports the results of our study [48]. Based on our results, we believe that aerobic and resistance exercise effectively reduce apelin concentration in obese middle-aged women. In addition, apelin response to human exercises was observed for the first time in Korea. On the contrary, the correlation between apelin-12 and apelin-36 by exercise has to be studied further.

As a result of 8 weeks of aerobic and resistance exercises in the present study, the AEG and the REG showed a decrease in weight, WC, BMI, and body fat, and apelin-12 levels decreased among obese middle-aged women in the AEG; this study was able to confirm that aerobic and resistance exercises had caused the decline in apelin-12 levels. Nevertheless, there is a limitation to its application as a best practice to reduce apelin levels for all obese women, since the study results were obtained from middle-aged women, who were more likely to experience sudden changes in hormones due to menopause and have the risk of exposure to metabolic syndrome. Accordingly, a wide range of studies need to be continued to examine changes in apelin levels depending on the exercise period, different types of exercises, age, and accompanying dietary changes.

## Conclusion

In this study, we investigated the effects of 8 weeks of aerobic or resistance exercise on physical composition and apelin-12 and apelin-36 concentrations in obese, middle-aged women. With our analysis, we derived the following conclusions. First, when changes in physical composition after 8 weeks of aerobic or resistance exercise were examined, both exercise types were effective for improving indicators for obesity, showing significant reductions in body weight, WC, BMI, and body fat. Second, aerobic and resistance exercise had a positive effect on improving indicators for metabolic syndrome, as demonstrated by significant decrease in blood apelin-12 concentration, which is closely correlated with indicators for metabolic syndrome.

Based on these results, 8 weeks of training in obese, middle-aged women resulted in decreases in weight, WC, BMI, and body fat in the AEG and REG and a decrease in apelin-12 concentration in the AEG. Therefore, previous exercise and apelin tests were conducted on subjects with diabetes or disease. However, in the present study, body composition change and apelin concentration were improved. Regular aerobic and resistance exercise training was correlated with a decrease in apelin concentration and a positive change in body composition such as body fat and WC, thereby lowering the risk of cardiovascular disease and metabolic syndrome as well as obesity.

Apelin, which is a recently identified adipokine, is involved in regulating insulin secretion and lipolysis, but there has been a lack of research examining changes and effects of apelin concentration via various pathways and types of exercise. Therefore, in future studies, it will be necessary to examine changes in apelin-12 and apelin-36 concentration in obesity, diabetes, and metabolic syndrome patients, after changes in physical composition via dietary regulation, a combination of exercise and dieting, or exercise only and to compare changes in physical composition and exercise performance of men and women of different age groups to examine whether there are any differences in the effects of apelin.

## Abbreviations

AEG: Aerobic exercise group
BMI: Body mass index
CG: Control group
REG: Resistance exercise group
WC: Waist circumference
WHO: World Health Organization
